# Identification and characterization of microRNAs in the pituitary of pubescent goats

**DOI:** 10.1186/s12958-018-0370-x

**Published:** 2018-05-25

**Authors:** Jing Ye, Zhiqiu Yao, Wenyu Si, Xiaoxiao Gao, Chen Yang, Ya Liu, Jianping Ding, Weiping Huang, Fugui Fang, Jie Zhou

**Affiliations:** 1 Anhui Provincial Laboratory of Animal Genetic Resources Protection and Breeding, College of Animal Science and Technology, Anhui Agricultural University, 130 Changjiang West Road, Hefei, 230036 Anhui China; 2 Anhui Provincial Laboratory for Local Livestock and Poultry Genetic Resource Conservation and Bio-Breeding, 130 Changjiang West Road, Hefei, 230036 Anhui China; 3 Department of Animal Veterinary Science, College of Animal Science and Technology, Anhui Agricultural University, 130 Changjiang West Road, Hefei, 230036 Anhui China

**Keywords:** Goat, MicroRNA, Pubescent, Pituitary

## Abstract

**Background:**

Puberty is the period during a female mammal’s life when it enters estrus and ovulates for the first time; this indicates that a mammal is capable of reproduction. The onset of puberty is a complex and tightly coordinated biological event; it has been reported that microRNAs (miRNAs) are involved in regulating the initiation of puberty.

**Methods:**

We performed miRNA sequencing on pituitary tissue from prepubescent and pubescent goats to investigate differences in miRNA expression during the onset of puberty in female goats. The target genes of these miRNAs were evaluated by GO enrichment and KEGG pathway analysis to identify critical pathways regulated by these miRNAs during puberty in goats. Finally, we selected four known miRNA and one novel miRNAs to evaluate expression patterns in two samples via qRT-PCR to validate the RNA-seq data.

**Results:**

In this study, 476 miRNAs were detected in goat pituitary tissue; 13 of these were specifically expressed in the pituitary of prepubescent goats, and 17 were unique to the pituitary of pubescent goats. Additionally, 73 novel miRNAs were predicted in these two libraries. 20 differentially expressed miRNAs were identified in this study. KEGG pathway enrichment analysis revealed that the differentially expressed miRNA target genes were enriched in pathways related to ovary development during puberty, including the GABAergic synapse, oxytocin signaling pathway, the cAMP signaling pathway, progesterone-mediated oocyte maturation. In this study, differential miRNA expression in the pituitary tissue of prepubescent and pubescent goats were identified and characterized.

**Conclusion:**

These results provide important information regarding the potential regulation of the onset of goat puberty by miRNAs, and contribute to the elucidation of miRNA regulated processes during maturation and reproduction.

**Electronic supplementary material:**

The online version of this article (10.1186/s12958-018-0370-x) contains supplementary material, which is available to authorized users.

## Background

MicroRNAs (miRNAs) are a class of non-coding RNAs that play a key roles in regulating gene expression during transcription and post-transcriptionally regulating protein expression [1]. MiRNAs are short, single-stranded RNA molecules that are approximately19–23 nucleotides in length [2]. Genes that are regulated by miRNAs account for 10–30% of all protein-coding gene [3]. MiRNAs have two canonical activities to implement gene regulation: the first way which is most effective useful in plants, is a miRNA to bind to a fully complementary sequence on a target mRNA and induces its cleavage [4, 5]. Another method of miRNA gene targeting is through incomplete matching of a miRNA to a partial complementary sequence on the 3′ untranslated region (3’ UTR) of its target mRNA, resulting in degradation of the mRNA and/or inhibition of protein translation [6]. MiRNAs play an important role in regulating many biological processes, including cell proliferation [7], apoptosis [8], cell differentiation [9], metabolism [10], hematopoiesis, and development [8]. Recent studies have shown that miRNAs play a direct role in apoptosis of bovine luteal regulation [11], suggesting that miRNAs are involved in reproductive regulation.

The pituitary is an important mammalian endocrine gland that composed of the adenohypophysis and neurohypophysis. Hormones produced and released by the pituitary affect many biological processes including animal growth, bone metabolism, and cell cycle activity [12]. Recently, studies have found that miR-26b plays an important role in pituitary development [13]. miR-15 and miR-16 are down-regulated in pituitary adenomas and are associated with secretion of p43 protein [14], suggesting a relationship between miRNAs and pituitary function.

Puberty is a critical stage of female goat development. It marks the first occurrence of ovulation and the onset of reproductive capability [15]. The mechanism of puberty onset is complex and thought to be associated with environmental factors, neuroendocrine factors, genetic factors, and interactions between these factors. The strongest factor that contributes to the onset of puberty is thought to be inheritable, as the development and timing of puberty are highly heritable. Genome wide association studies (GWAS) have identified many loci which may affect the timing and development of puberty [16]. GWAS have disclosed that variants in/near LIN28b influence both adult height and onset of menarche [17]. LIN28 and LIN28b are related RNA-binding proteins, they bind to the terminal loops of let-7 miRNA family, inhibiting the processing of let-7 family members into mature miRNA [18]. These reports indicate that puberty is likely closely regulated by miRNAs, and that miRNA are likely involved in the onset of puberty in mammals.

We hypothesized that the onset of goat puberty is also regulated by miRNAs, and that critical pituitary functions during puberty may be regulated by miRNA. The expression profile of miRNAs in the pituitary of pubescent goat remains unknown. In this study, we applied Solexa sequencing and investigated the expression of miRNAs in prepubescent and pubescent goats to explore the relation of miRNAs with the onset of puberty.

## Methods

### Pituitary collection and total RNA isolation

Three pubescent Anhuai female goats, aged 4.5–5 months and weighing 17.43 ± 1.63 kg, and three prepubescent aged 2.5–3 months and weighing 9.6 ± 2.36 kg, were used in this study. Pubescent goat was identified via the change in vaginal and ovarian physiology (Additional file 1), hormone profiles (Additional file 2) and rams test conditions [19]. Briefly, rams test conditions is use a healthy ram to test that whether female goats were in estrus. A piece of cloth was tied on the abdomen of the ram to prevent mating while testing. Mount behaviour would occur when the female goats were in estrus. Rams test conditions were performed twice daily at 08:00 and 16:00. The cunnus of pubescent goats became inflamed, and histological observation identified some mature follicles in the ovaries(Additional file 1). Pituitary glands were collected from pubescent (*n* = 3) and prepubescent (n = 3) goats [20, 21] after animals were anesthetized with injection of 0.1 ml xylazine hydrochloride (Muhua China, Lot number 150804) before sacrificed. And the surgery for removing the pituitary is feferred the study of Bjarkam et al. [22]. The collected tissues were immediately placed in liquid nitrogen and stored at − 80 °C. Total RNA was isolated using TRIzol (Invitrogen, Carlsbad, CA, USA) according to the manufacturer’s protocol. RNA integrity for sequencing was assessed using the RNA Nano 6000 Assay Kit and a Agilent Bioanalyzer 2100 system (Agilent Technologies, CA, USA). The concentration of total RNA for qRT-PCR was quantifiedc at 260 nm with a NanoDrop spectrophotometer (ND-2000, USA). The quality of total RNA for qRT-PCR was assessed by agarose gel electrophoresis.

### Small RNA library construction and sequencing

A total of 3 μg total RNA per sample was used as input material for the small RNA (sRNA) library construction. Sequencing libraries were generated using NEBNext® Multiplex Small RNA Library Prep Set for Illumina® (NEB, USA.) following manufacturer’s recommendations. Total RNA was used as the starting sample, the sRNA ends are directly connected with the adapter, followed by reverse transcription synthesis into cDNA. DNA fragments of 140-160 bp were separated by PAGE gel electrophoresis, and the cDNA library was recovered. Finally, library quality was assessed on the Agilent Bioanalyzer 2100 system using DNA High Sensitivity Chips.

### Sequence analysis

Clean sequencing reads were obtained by removing from the raw data reads containing poly-N, with 5′ adapter contaminants, without 3′ adapters or the insert tag, containing poly A, T, G, or C, low quality reads, and reads shorter than 18 nt. After read clean up, the high-quality reads were mapped to a reference sequence by Bowtie [23] without mismatch to analyze their expression and distribution on the reference sequence.

To remove tags originating from protein-coding genes, repeat sequences, rRNA, tRNA, snRNA, and snoRNA, sRNA tags were mapped to the RepeatMasker, Rfam database. The clean reads were compared to the miRNA precursor/mature miRNA of all animals in miRBase 21.0, and show the sequence and count of miRNA families (not species-specific) that can be found in the samples. The characteristics of the hairpin structure of miRNA precursors was evaluated to predict novel miRNAs.

### Differential expression analysis

In order to find out the differentially expressed miRNAs between pituitary tissue from prepubescent and pubescent goats, expression data were Log2-transformed ratio figure and plotted on a scatter plot. Briefly, the procedures were as follow: (1) miRNA expression from the two libraries was normalized to obtain the expression of transcript per million reads (TPM). Normalization formula: Normalized expression = mapped readcount/Total reads*1 × 10^6; (2) Calculate fold-change and *P*-value from the normalized expression. P-value was adjusted using qvalue. Qvalue< 0.01 and |log2(foldchange)| > 1 was set as the threshold for significantly differential expression by default. Finally, generate the Log2-ratio figure and Scatter Plot.

When the normalized expression of a miRNA was zero between two libraries, its expression value was adjusted to 0.01 (as 0 cannot be plotted on a log plot). If the normalized expression of a certain miRNA in two libraries was all lower than 1, further differential expression analysis was conducted without this miRNA.

### GO enrichment and KEGG pathway analyses

Gene Ontology (GO; http://www.geneontology.org) is an international standard classification system for gene function. After selecting miRNA target genes, the distribution of the target genes among biological pathways/functions in Gene Ontology will clarify the biological differences between the samples based on gene function. Using this method, first candidate target genes are mapped to the GO terms (biological functions) in the database (http://www.geneontology.org), the number of genes in every term is calculated, and a hypergeometric test is performed to identify significantly enriched GO terms in the target gene candidate list out of the background of the reference gene list.

The Kyoto Encyclopedia of Genes and Genomes (KEGG) database is a public database of pathway data, and is a resource for understanding high-level functions and processes active in a biological system [24]. KEGG pathway analysis identifies significantly enriched metabolic pathways or signal transduction pathways enriched in target gene candidates compared to a reference gene background, using the hypergeometric test.

### Quantitative real-time PCR

We extracted the total RNA from the pituitary, then, the quality and concentration of the total RNA was assessed. Samples demonstrating satisfactory RNA quality were selected for further analysis. That is, in the agarose gel electrophoresis, the RNA showed three bands, they are 28 s, 18 s and 5 s, respectively (Additional file 3). And the concentration of total RNA for each example was over 555 ng/μl (Additional file 4), which showed low degradation.

In order to validate the RNA-seq data, we randomly selected four known miRNA and one novel miRNAs to evaluate expression patterns in prepuberty and puberty via qRT-PCR. Another three goats were used in each group and the qRT-PCR experiments were repeated three times per sample, and all of the reactions were carried out in triplicate. We used Primer 5 software to design primers online and evaluated specificity using BLAST at NCBI. The list of the forward primer and universal miRNA qPCR primer sequences are shown in Table 1 and U6 was housekeeping gene. We used TransScript^®^ Green miRNA Two-Step qRT-PCR SuperMix(TransScript, AQ202, China) to perform reverse transcription and qRT-PCR. The reverse transcription reaction system and program are shown as below: (1)Mixed 500 ng total RNA, 1 μl TransScript^®^ miRNA reverse transcription(RT) enzyme mix, 2× TS miRNA reaction mix and RNase-free water to 20 μl, and then incubated the mixture at 37 °C for 1 h followed inactivated the RT enzyme mix at 85 °C for 5 s. The cDNA obtained after reverse transcription is diluted 10-fold before qPCR. The PCR mixture included 2 μl of cDNA for every miRNA, 0.4 μl forward primer(10Um), 0.4 μl universal miRNA qPCR primer(10uM), 10 μl 2 × TransScript^®^ tip g reen qPCR supermix, 0.4 μl passive reference dye(50×) and ddH_2_O to 20 μl. The PCR conditions were as follows: initial denaturation at 94 °C for 30 s, followed by 40 cycles of 94 °C for 5 s, 60 °C for 15 s, and 72 °C for 10 s, and a terminal hold at 4 °C. We collected the cycle threshold (Ct) from each reaction, and the expression level of each gene was evaluated using the 2^-ΔΔCT^ method.

**Table 1:** Primer sequences for qRT-PCR

The name of primer	Sequence,5′-3’
chi-miR-543-3p	AAACATTCGCGGTGCACTTCT
chi-miR-493-5p	TTGTACATGGTAGGCTTTCATT
chi-miR-335-3p	TTTTTCATTATTGCTCCTGACC
chi-miR-411a-3p	TATGTAACACGGTCCACTAAC
novel_128	TAATCTCAGCTGGCAACTGTGA
U6	GGCAAGGATGACACGC
Universal miRNA qPCR Primer	GATCGCCCTTCTACGTCGTAT

### Statistical analysis

We used the statistical package R statistical software (R, Auckland, NZL) for further analysis of RNA-seq data. R was used for graphical representations, as well as to correct for to multiple testing and *P*-value corrections. We analyzed the qRT-PCR data by SPSS 17.0 software package (SPSS, Chicago, IL, USA). Data were expressed as mean ± standard error, with *P* < 0.05 indicating significant difference. Graphpad 5.0 was used to draw figures.

## Results

### Overview of RNA sequencing data

**Fig. 1:**
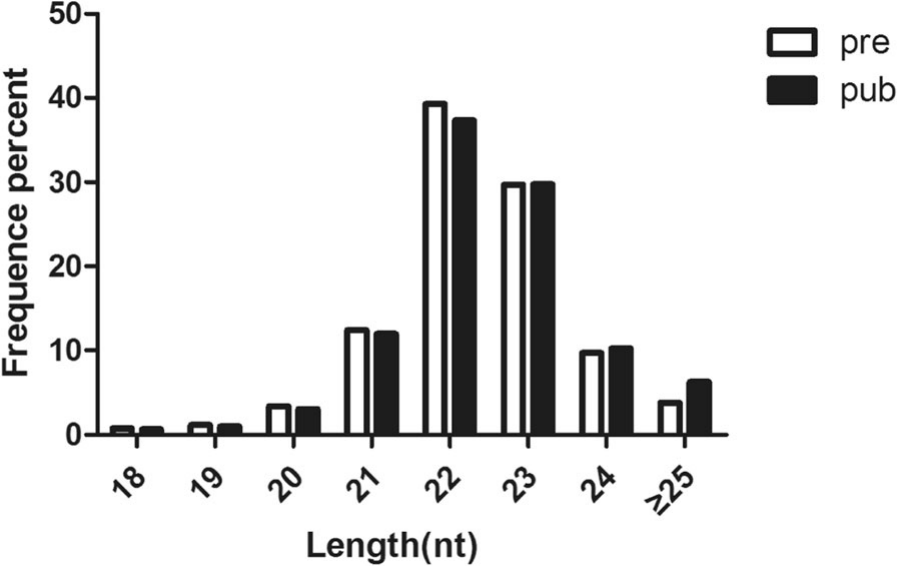
Frequency distribution of sRNA sequence lengths. Pre: prepubescent pituitary library; Pub: pubescent pituitary library

In order to identify miRNAs that are differentially expressed the pituitary of pubescent and prepubescent goats, two sRNA libraries were constructed by Solexa sequencing. The error rate of the sequencing data from these two libraries is 0.01%, and the Q30 ≥ 97.8%, indicating that the sequencing data is high quality and suitable for this study. A total of 11,793,577 reads and 10,676,789 reads were acquired from the pituitary libraries of pubescent and prepubescent goats, respectively. After discarding the sequences that are below background, over 86% sRNAs were mapped to the reference genome in the two libraries (Table 2). Subsequently, all identical sequence reads were classified as groups, and 365,515 and 296,547 unique sequences were obtained. We then chose a specific sRNA length range from the clean reads and calculated the length distribution of sRNA (Fig. 1). The majority of the sRNAs are between 21 and 24 nt in size. Sequences of 22 nt in length, the traditional size of Dicer-derived products [25], accounted for 39.25 and 37.3% of the total sequence reads in the pituitary libraries from pubescent and prepubescent goats, respectively.

**Table 2:** The quality of library sequences by Solexa sequencing

Type	Pub		Pre	
	Counts	Percent	Counts	Percent
Total reads	11,793,577	100%	10,676,789	100%
N% > 10%	124	0.00%	125	0.00%
Low quality	3434	0.03%	2956	0.03%
5′ adapter contamine	165	0.00%	93	0.00%
3 adapter null or insert null	234,299	1.99%	184,344	1.73%
With polyA/T/G/C	5158	0.04%	4261	0.04%
Clean reads	11,550,397	97.94%	10,485,010	98.20%

*Pre* preuberty; *Pub* puberty

**Fig. 2:**
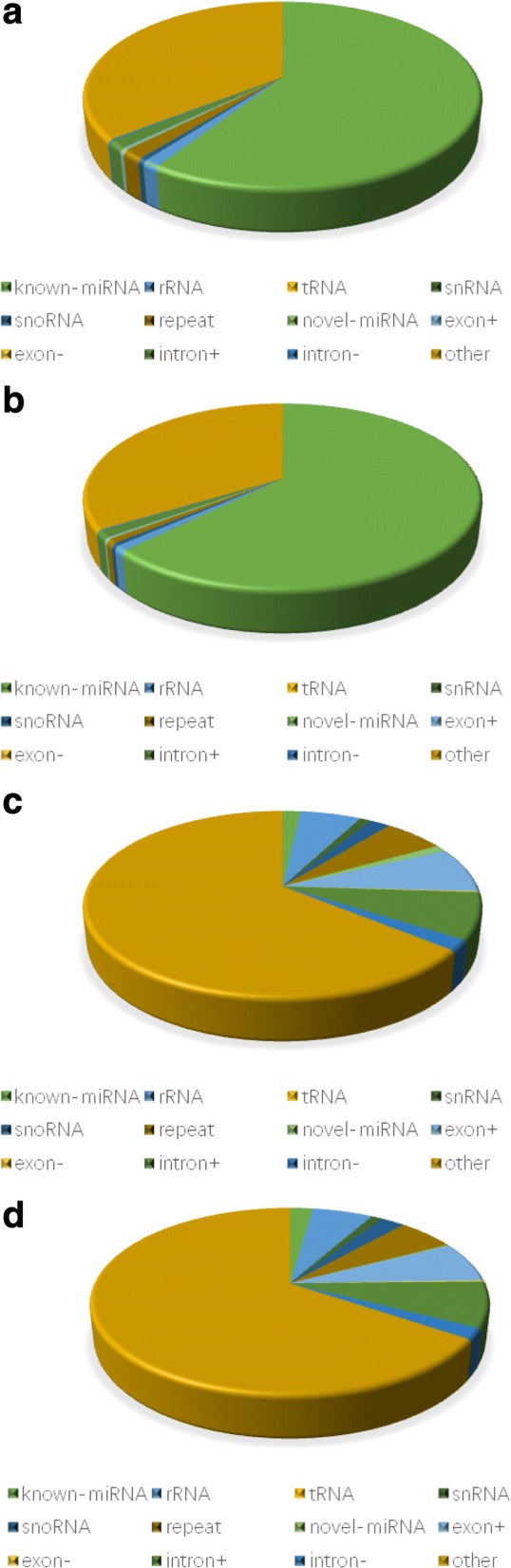
Composition of small RNA classes from Solexa sequencing. **a** Total number of reads in the pubescent goat pituitary library. **b** Total number of reads in the prepubescent goat pituitary library. **c** Total number of unique sequences in the pubescent goat pituitary library. **d** Total number of reads in the prepubescent goat pituitary library. Pre: prepubescent pituitary library; Pub: pubescent pituitary library

In order to analyze the sRNA expression and distribution based on the reference sequence, the sRNAs were mapped to the reference sequence by Bowtie [23] without mismatch, and 8,823,349 and 9,765,115 sRNAs were obtained. The remaining reads were compared with the RepeatMasker, Rfam database to remove possible mRNA, rRNA, tRNA, snRNA, snoRNA and repeat sequences. However, some sRNA tags may be mapped to more than one category. To make every unique sRNAs mapped to only one annotation, we followed the following priority rule: rRNAetc (Genbank > Rfam) > known miRNA > repeat > exon > intron [26]. All of the clean reads were divided into the following categories: exon_sense, exon_antisense, intron_sense, intron_antisense, miRNA, rRNA, repeat, scRNA, snRNA, snoRNA, srpRNA, tRNA, unknown (sequences not mapped to any known reference databases). The composition of the RNA classes in each library is shown in Fig. 2. The proportion of total rRNA is indicative of sample quality; for a high quality sample it should be less than 60% in plant samples [27] and 40% in animal samples (unpublished data by BGI). The proportion of total rRNA was 1.16 and 0.87% in the pituitary libraries of pubescent and prepubescent goats, respectively, indicating that the pituitary RNA samples collected were of high quality. In the clean reads from the pituitary libraries of pubescent and prepubescent goats, 8,823,349 reads (account for 86.84%) and 9,765,115 reads (account for 86.64%), respectively were mapped to the goat reference genome (Additional file 5). Known miRNAs accounted for 62.57 and 59.16% of the total clean reads, and accounted for 2.23 and 1.76% of the unique reads in the pituitary sRNA libraries of pubescent and prepubescent goats, respectively (Fig. 2). The analysis of these two libraries suggests that miRNA sequences are enriched among the sRNA libraries.

### Known miRNAs

In order to identify known miRNAs in goat pituitary, the dataset was compared to known miRNAs (miRNA precursors and mature miRNAs) in miRBase21.0. A total of 3669 and 3781 unique sequences in the pituitary libraries of pubescent and prepubescent goats,were mapped to known miRNAs in miRBase 21.0, respectively. Our results showed a total of 403 mature miRNAs, 253 miRNA hairpins, 7450 unique sRNA, and 11,297,037 total sRNA were obtained (Table 3).

**Table 3:** Statistics of known miRNA identified in pituitary of prepubescent and pubescent goats

Types	Total	Pub	Pre
Mapped mature	403	397	396
Mapped hairpin	253	253	254
Mapped unique sRNA	7450	3669	3781
Mapped total sRNA	11,297,037	5,776,657	5,520,380

*Pre* preuberty; *Pub* puberty

### Identification of potential novel miRNAs

The presence of a hairpin RNA structure, characteristic of a miRNA precursor, can be used to predict novel miRNAs. Novel miRNAs were predicted by mapping precursor sequences to goat the genome by integrated miREvo [28] and miRDeep2 [29] miRNA prediction software. We detected 73 potential novel miRNAs (Table 4). Novel miRNAs were predicted by exploring the secondary structure, predicting the Dicer cleavage site and binding energy.

**Table 4:** Statistics of the predicted novel miRNAs mapping to small RNAs

Types	Total	Pub	Pre
Mapped mature	73	66	63
Mapped star	33	28	29
Mapped hairpin	79	72	71
Mapped unique sRNA	603	302	301
Mapped total sRNA	23,994	13,624	10,370

*Pre* preuberty; *Pub* puberty

### Differential expression of miRNA in the pituitary of pubescent and prepubescent goats

**Fig. 3:**
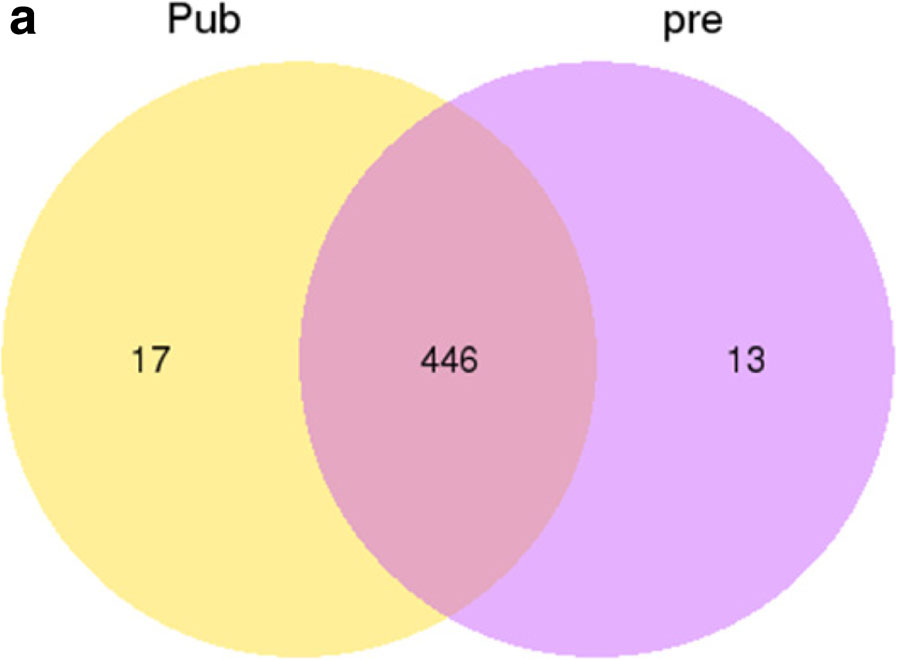
Differentially expressed miRNAs in the two goat pituitary libraries. **a** Venn diagram displaying the distribution of 476 unique miRNAs in the pubescent goat pituitary (left, yellow circle) and prepubescent goat pituitary libraries (right, purple circle). The overlapping region indicates co-expressed unique miRNAs. Pre: prepubescent pituitary library; Pub: pubescent pituitary library

As shown in Fig. 3, 476 unique miRNA were analyzed from the pituitary libraries of pubescent and prepubescent goats (Additional file 6). Among them, 446 miRNAs were co-expressed in both libraries, while 17 miRNAs were specifically expressed in pubescent goat pituitary and 13 miRNAs were specifically expressed prepubecent goat pituitary.

Using a volcano plot, the overall distribution of the miRNAs can be determined. Significatly differentially expressed miRNAs are screened based on two factors: Fold change and corrected level (*padj* / *qvalue*). After repetition of the biological samples, differentially expressed miRNAs were screened as: *padj* < 0.05. Twenty miRNA were significantly differentially expressed, including ten over-expressed miRNAs, and ten under-expressed miRNA (Additional file 7).

### miRNA target gene prediction

Prediction of miRNA target genes performed by miRanda. In the two libraries, 653,807 target sites in 25,619 target genes were predicted for the 403 known miRNAs 126,299 target sites in 25,015 target genes were predicted for the 73 novel miRNAs (Additional file 8).

### Gene ontology (GO) enrichment and KEGG pathway analysis of miRNA target genes

**Fig. 4:**
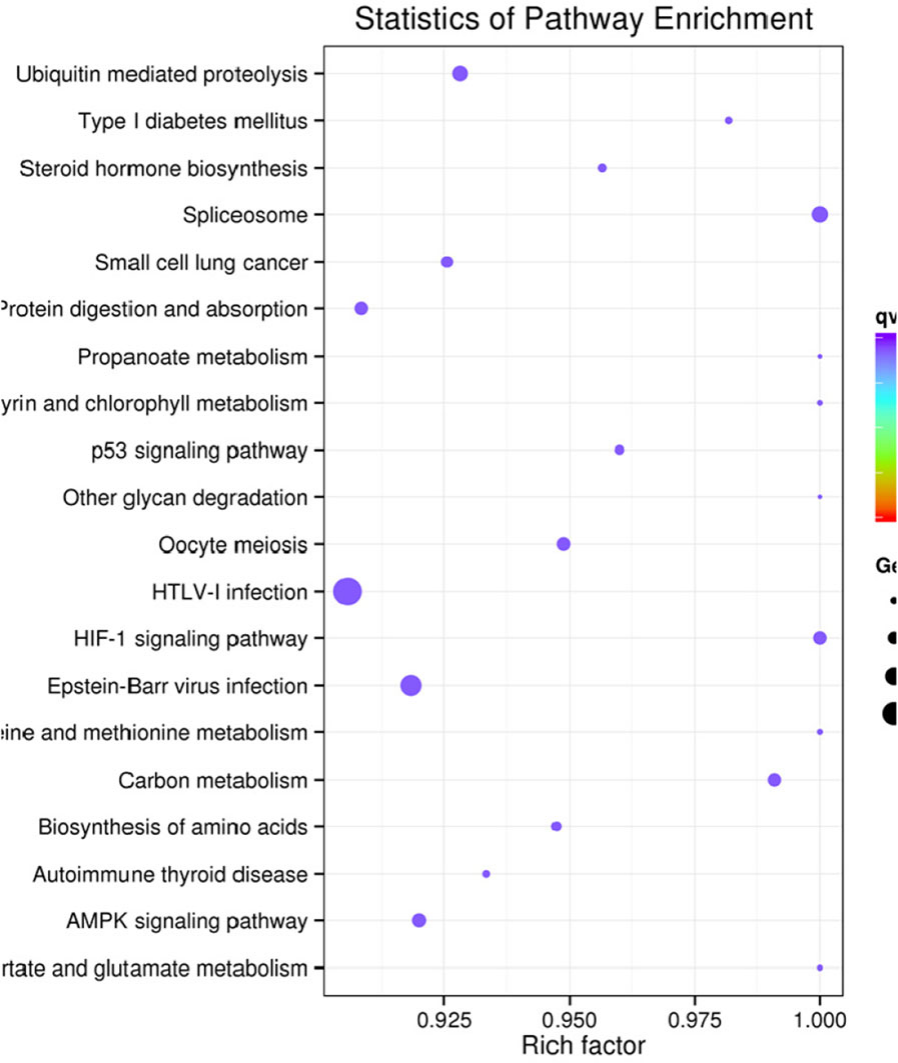
The vertical axis represents the pathway name, and the horizontal axis represents the enrichment factor. The size of the dot indicates the number of candidate target genes in the pathway, and the color of the dot corresponds to the different Q value range. Pre: prepubescent pituitary library; Pub: pubescent pituitary library

In this study, the candidate target genes for differentially expressed miRNAs was used for the GO enrichment assessment to predict biological functions. Statistical analysis of the significantly enriched number of genes in each term are indicated in Fig. 4.

KEGG pathway analysis showed that 273 pathways were involved in the candidate genes of miRNAs present in the pituitary tissue of prepubescent and pubescent goats. The enriched terms were mainly focused on the olfactory transduction signaling pathway. The results of KEGG pathway analysis also indicated seveal enriched terms were involved in puberty, such as Vasopressin-regulated water reabsorption, Glutamatergic synapse, Oxytocin signaling pathway, cAMP signaling pathway, Progesterone-mediated oocyte maturation, and the GnRH signaling pathway (Fig. 4, Table 5).

**Table 5:** List of the candidate target genes with significantly enriched pathways and functions

Term	Sample number	Background number	*P*-value	Corrected *P*-value
Vasopressin-regulated water reabsorption	14	45	0.412	0.951
Glutamatergic synapse	35	114	0.342	0.951
Oxytocin signaling pathway	53	155	0.117	0.951
cAMP signaling pathway	66	213	0.249	0.951
Progesterone-mediated oocyte maturation	24	84	0.498	0.951
GnRH signaling pathway	24	88	0.578	0.951

### Quantitative RT-PCR validation of miRNA expression

**Fig. 5:**
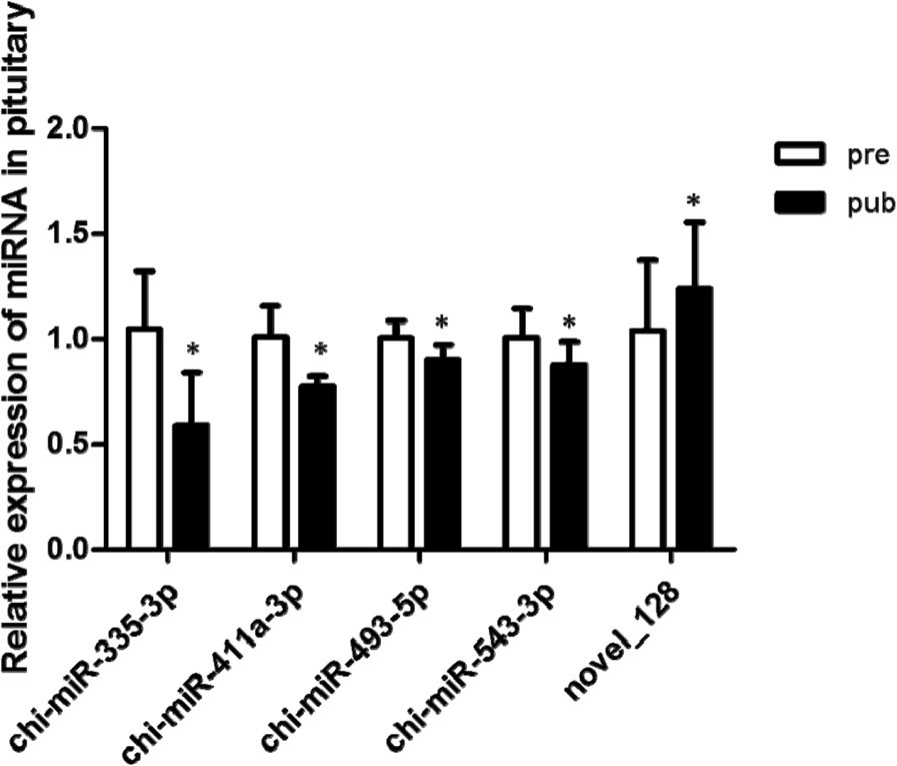
qRT-PCR validation of Solexa sequencing. Pre: prepubescent pituitary; Pub: pubescent pituitary

The expression of 5 different miRNA from the pituitary of pubescent and prepubescent goats were selected randomly for expression validation using qRT-PCR. qRT-qPCR analysis with U6 as housekeeping gen indicated that the expression of chi-miR-335-3p, chi-miR-493-5p, chi-miR-543-3p and chi-miR-411a-3p had decreasing trends (*P* < 0.05), and the expression of novel_128 was increased (*P* < 0.05) in the pubescent goats compared to prepubescent goats (Fig. 5); these data were consistent with the Solexa sequencing results, which indicates that these miRNAs may be involved in regulating the onest of puberty.

## Discussion

The normal and regular onset of puberty and sexual maturation is of critical importance to the reproductive performance of goats. Breeding goats early in pubescence can not only reduce feeding costs and enhance the utilization of females, but also can shorten the interval between female generations and can accelerate selective breeding methods. Therefore, there are a focus on molecular-assisted breeding techniques and miRNAs in breeding research [30–34].

When we initially reported on miRNA expression in the pituitary of pubescent goats, samples from individual animals were mixed, according to previous papers [21, 32]. Mixing of samples for sequencing can impair the detection of low abundance genes [20, 21]. Nevertheless, the qRT-PCR validation of differential miRNA expression from pituitary tissue of individual animals that we report here indicates results of the Solexa sequencing are reliable.

In the present study, sRNAs in the pituitary tissues of prepubescent and pubescent goats were sequenced by Illumina Solexa technology. We analyzed the differentially expressed miRNAs, predicted the novel miRNAs, and performed GO enrichment and KEGG pathway analysis of target genes from these two miRNA libraries.

Sequencing revealed that the sRNA sequence lengths in the sRNA libraries were between 20 and 24 nt; the most abundant sRNA length from the total sRNA sequences was at 22 nt, which is the classical length of goat miRNAs. The distribution of length in sRNAs of goats was similar to previous studies indicating the distribution of sRNA in cattle, pigs, and chickens [34–37]. The peak length of what sRNA that was obtained by high-throughput sequencing was at 24 nt [38], indicating that there are significant differences in the distribution of miRNA between species. The localization of miRNAs may be associated with miRNA function, and studies of miRNA localization can provide a rich blueprint for the elucidating the architecture of the goat genome [39, 40]. In this study, 403 known mature miRNAs were found by mapping sRNAs to the known miRbase. According to the pre-miRNA structure in the reference genome sequence, 73 new miRNAs were predicted; this greatly enriches our understanding of the goat miRNA transcription, and lays a foundation for further study on the role of mRNA and miRNA regulatory networks in goats.

Using Solexa sequencing, we identified 463 and 459 unique miRNAs in the pituitary RNA libraries from pubescent and prepubescent goats, respectively. Compared with previous studies [41], we identified more goat miRNA using our method. This may be influenced by the fact that we compared the sequences of our clean reads to miRBase 21.0, which has additional miRNA species compared to earlier miRBase releases. We also we compared sRNA sequences to all animal miRNAs, including all species of miRNAs, thereby increasing the number of miRNA sequences that was available to compare.

The known miRNA and novel miRNA were analyzed for differential expression levels: twenty miRNA genes were significantly differentially expressed, evaluated both by fold change and corrected significance level (q < 0.05); these differential miRNA genes were novel_128, which was over-expressed, and chi-miR-335-3p, chi-miR-411a-3p, chi-miR-493-5p, and chi-miR-543-3p, which were under-expressed. These four known miRNA were also found in Saanen dairy goat [42], but the function in reproductive or physiological still remain unknown until now. Chi-miR-9-5p and chi-miR-30f-5p are two differentially expressed genes in the present study, and similarly, they are also differentially expressed in the ovaries of Jining gray and Laiwu black goats [43]. Homologues of differentially expressed miRNAs play a role in various cellular activities. For instance, chi-miR-9-3p has been shown to be an important regulator of osteoblast differentiation in mouse iPS cells and also targets β1 integrin to sensitize claudin-low breast cancer cells to MEK inhibition [44, 45]. The chi-miR-9-5p processed by the same precursor may be involved in the regulation of puberty initiation in this study. Two highly expressed differential miRNAs (miR-10b and miR-99a) identified in the present study are also highly expressed in the ovaries of goats, pigs and other animal species as reviewed by Li et al. [46]. MiR-99a is one of the most important miRNA populations in the ovary of mammals [32, 43, 47]; it induces G1-phase cell cycle arrest and inhibits tumorigenesis, which may play a decisive part in normal ovarian function [48, 49]. MiR-335 is involved in rat epididymal development via regulation of the RAS p21 protein activator 1 [50]. In human stem cells, miR-335 regulates cell proliferation, migration and differentiation [51, 52] and is involved in the inhibition of tumor reinitiation [53]. In this study, we found that miR-335 is under-expressed in pubescent goat pituitary, which indicates that miR-335 may be related to the regulation of goat empathema. There are few other reports on the relationship between miRNA expression and puberty. KEGG pathway annotations of miR-34c show that most of its target genes are involved in cancer signaling pathways and in actin cytoskeleton KEGG terms. MiR-34c was reported to be important for self-renewal of spermatogonial stem cell and spermatogenesis [54], was found to regulate differentiation of mouse embryonic stem cells into male germinal cells through RARg [55], was shown to operate downstream of p53 to induce apoptosis in germ line stem cells (mGSCs) of male dairy goats [56]. In this study, we found that miR-34c is a up-regulated in puberty, indicating that miR-34c may be involved in the regulation and control of goat empathema.

MiRNA also plays an important part in central neuroendocrine control of the reproductive system. MiRNAs active in the hypothalamic GnRH network. For instance, previous study shows that miRNA-155 and miRNA-200/429 are key components of a complex developmental switch that control the GnRH promoter activity, and its function is necessary for initiation of puberty and reproduction in animals [57]. Blocking the expression of miR-132/212 impairs the up-regulation of FSH secretion by GnRH [58]. These findings indicates that miRNA could participate in the process of GnRH-regulated gonadotropin secretion, which directly affect the onset of puberty in animals.

We used TargetScan to predict the gene targets of known and novel miRNAs, and we evaluated these target genes with GO enrichment and KEGG functional analysis. In the KEGG functional enrichment, candidate target genes are involved in pathways related to puberty, including Vasopressin-regulated water reabsorption, Glutamatergic synapse, Oxytocin signaling pathway, cAMP signaling pathway, Progesterone-mediated oocyte maturation, and the GnRH signaling pathway. It is worth to noting that some target genes of miRNAs that were not differentially expressed in the pituitary of prepubescent and pubescent goats are also involved in puberty development-related networks. In summary, the enrichment of these target gene pathways may provide a reference for future research in this field.

In summary, we identified 446 miRNA that were co-expressed, and 13 and 17 miRNA that were specifically expressed in the pituitary of prepubescent and pubescent goats, respectively. We predicted 73 novel miRNA were from the two pituitary RNA libraries. We identified four miRNAs that were differentially expressed between prepubescent and pubescent goat pituitary, including three that were over-expressed and one that was under-expressed. KEGG pathway enrichment analysis showed that the target of miRNAs were significantly enriched in pathways related to puberty. These results provide important information regarding the potential regulation of the onset of goat puberty by miRNAs, and contribute to the elucidation of miRNA regulated processes during maturation and reproduction.

## Conclusion

Our results demonstrate that different expression of miRNA occur from prepuberty to puberty in goat. The results provide important information for the potential regulation of the onset of goat puberty and contribute to elucidate the processes of miRNA regulation during maturation and reproduction.

## Additional files

Additional file 1: Character of prepuberty and puberty goat. A: Vulva of goat in prepuberty. B: Vulva of goat in puberty. C: Ovarian of goat in prepuberty. D: Ovarian of goat in puberty. (DOCX 1429 kb)

Additional file 2: Serum E2 and P4 levels the development of puberty in Anhuai goat (Mean ± SE). Note:Means with the different superscripts within the same column differ significanly(*P* < 0.05) (DOCX 15 kb)

Additional file 3: RNA quality. (DOCX 56 kb)

Additional file 4: The concentration of total RNA. Pre: prepubescent sample; Pub: pubescent sample. (DOCX 14 kb)

Additional file 5: Reads mapped to the goat reference genome in pubescent and prepubescent goat pituitary libraries. (XLS 75 kb)

Additional file 6: Unique sequences from the pituitary libraries of pubescent and prepubescent goat pituitary. (XLSX 10 kb)

Additional file 7: Significantly differentially expressed miRNAs in pubescent and prepubescent goat pituitary. (XLSX 10 kb)

Additional file 8: 73 novel miRNAs identified from pubescent and prepubescent goat pituitary. (XLS 30 kb)

## Abbreviations

GO: Gene ontology
GWAS: Genome wide association studies
KEGG: Kyoto encyclopedia of genes and genomes
