# Supplementary figures and images for: Identification and characterization of microRNAs in the pituitary of pubescent goats
Source: Reprod Biol Endocrinol. 2018 May 25;16:51. doi: 10.1186/s12958-018-0370-x (PMC5970454; doi:10.1186/s12958-018-0370-x)

Additional file 1


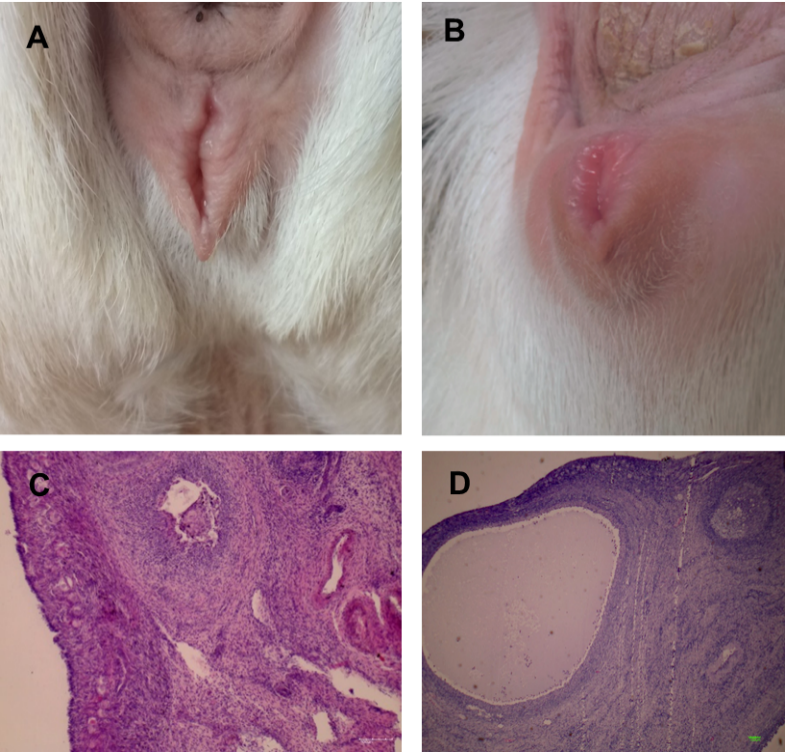
.

Supplement: Supplementary file 1 — Character of prepuberty and puberty goat. A: Vulva of goat in prepuberty. B: Vulva of goat in puberty. C: Ovarian of goat in prepuberty. D: Ovarian of goat in puberty. (DOCX 1429 kb) [file 12958_2018_370_MOESM1_ESM.docx]

Additional file 3


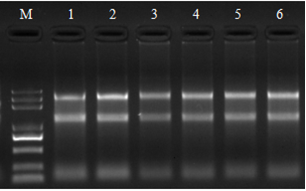

Supplement: Supplementary file 3 — RNA quality. (DOCX 56 kb) [file 12958_2018_370_MOESM3_ESM.docx]
