# Supplementary material for: Identification and characterization of microRNAs in the pituitary of pubescent goats
Source: Reprod Biol Endocrinol. 2018 May 25;16:51. doi: 10.1186/s12958-018-0370-x (PMC5970454; doi:10.1186/s12958-018-0370-x)
Supplement: Supplementary file 2 — Serum E2 and P4 levels the development of puberty in Anhuai goat (Mean ± SE). Note:Means with the different superscripts within the same column differ significanly(P < 0.05) (DOCX 15 kb) [file 12958_2018_370_MOESM2_ESM.docx]

Additional file 2

| Age(M) | E_2_(ng/L) | P_4_(pmol/L) | E_2_/P_4_ |
| --- | --- | --- | --- |
| 2.5 | 11.57±3.47 ^a^ | 1284.66±145.33 ^b^ | 0.009 |
| 2.75 | 16.91±4.85 ^b^ | 1072.72±264.72 ^a^ | 0.016 |
| 3 | 13.68±2.49 ^a^ | 862.60±144.96 ^a^ | 0.016 |
| 4.5 | 8.96±2.30 ^c^ | 1029.53±184.68 ^a^ | 0.009 |
| 4.75 | 8.85±1.28 ^c^ | 1068.10±194.21 ^a^ | 0.008 |
| 5 | 10.59±4.90 ^c^ | 1031.40±127.75 ^a^ | 0.010 |
