# Supplementary material for: Identification and characterization of microRNAs in the pituitary of pubescent goats
Source: Reprod Biol Endocrinol. 2018 May 25;16:51. doi: 10.1186/s12958-018-0370-x (PMC5970454; doi:10.1186/s12958-018-0370-x)
Supplement: Supplementary file 4 — The concentration of total RNA. Pre: prepubescent sample; Pub: pubescent sample. (DOCX 14 kb) [file 12958_2018_370_MOESM4_ESM.docx]

Additional file 4

| Sample | Concentration(ng/μl) | 260/280 | 260/230 |
| --- | --- | --- | --- |
| Pre-1 | 864.3 | 2.01 | 2.01 |
| Pre-2 | 1623.2 | 2.00 | 2.05 |
| Pre-3 | 2115.6 | 2.04 | 2.02 |
| Pub-1 | 1828.4 | 2.01 | 2.00 |
| Pub-2 | 1573.8 | 1.99 | 2.05 |
| Pub-3 | 1272.9 | 2.02 | 2.05 |
